# Bovine NK-lysin-derived peptides have bactericidal effects against *Mycobacterium avium* subspecies *paratuberculosis*

**DOI:** 10.1186/s13567-021-00893-2

**Published:** 2021-01-21

**Authors:** Rohana P. Dassanayake, Taylor L. T. Wherry, Shollie M. Falkenberg, Timothy A. Reinhardt, Eduardo Casas, Judith R. Stabel

**Affiliations:** 1 Ruminant Diseases and Immunology Research Unit, National Animal Disease Center, Agricultural Research Service, United States Department of Agriculture, Ames, IA USA; 2 Department of Veterinary Pathology, College of Veterinary Medicine, Iowa State University, Ames, IA USA; 3 Infectious Bacterial Diseases Research Unit, National Animal Disease Center, Agricultural Research Service, United States Department of Agriculture, Ames, IA USA

**Keywords:** Antimicrobial peptides, Amps, Bovine NK-lysins, bNK2A, Johne’s disease, MAP, *Mycobacterium avium* subspecies *paratuberculosis*, Propidium monoazide-based viability qPCR

## Abstract

Infection with *Mycobacterium avium* subspecies *paratuberculosis* (MAP) is complex, but little is known about the role that natural killer (NK) cells play. In the present study, four bovine NK-lysin peptides were synthesized to evaluate their bactericidal activity against MAP. The results demonstrated that bNK-lysin peptides were directly bactericidal against MAP, with bNK1 and bNK2A being more potent than bNK2B and bNK2C. Mechanistically, transmission electron microscopy revealed that the incubation of MAP with bNK2A resulted in extensive damage to cell membranes and cytosolic content leakage. Furthermore, the addition of bNK2A linked with a cell-penetrating peptide resulted in increased MAP killing in a macrophage model.

## Introduction, methods, and results

Infection with *Mycobacterium avium* subspecies *paratuberculosis* (MAP), an intracellular pathogen, causes chronic enteritis in ruminants, also known as Johne’s disease [1]. Transmission is primarily faecal-oral, and in the initial stages of infection, MAP is taken up by resident macrophages within the small intestine. The thick, waxy cell wall of MAP is composed of more than 60% lipids, leading to a high level of resistance to heat, environmental conditions, chemical treatment and antibiotics [2–5]. This complex mycobacterial cell wall makes it possible to survive the killing mechanisms of macrophages, such as lysosomal enzymes and reactive oxygen metabolites, allowing them to survive and replicate inside macrophages [6, 7].

Antimicrobial peptides (AMPs) or host defence peptides are a diverse group of molecules that are produced by all living organisms as a part of the innate immune system [8, 9]. Unlike antibiotics, most AMPs are effective against a broad spectrum of pathogens ranging from viruses to parasites. In general, AMPs are oligopeptides composed of varying numbers of amino acids but are typically 5–50 residues long [8, 10]. Bovine NK-lysins (bNK-lysins) are antimicrobial proteins produced by T lymphocytes and natural killer (NK) cells [11]. bNK-lysins are structurally and functionally similar to the well-characterized proteins human granulysin and porcine NK-lysin [11–14]. Granulysin and NK-lysins are found in the granules of human, porcine and bovine cytotoxic T-cells (CTLs) and NK cells [12, 14, 15]. In general, granulysin and NK-lysins are synthesized as precursor proteins (~15 kDa), and both ends are cleaved to produce an active protein (~9 kDa). The larger precursor protein is secreted by CTLs and NK cells, while the mature form is stored and released from cytotoxic granules to target bacteria and infected cells.

One of the earliest reports of the antimycobacterial activity of granulysin demonstrated that CTLs killed intracellular pathogens such as *M. tuberculosis* in a granule-dependent manner [12]. This was followed by the finding that active regions of porcine NK-lysin and human granulysin peptides were effective in killing *M. tuberculosis*, as well as *Pseudomonas aeruginosa*, *Staphylococcus aureus*, and *Escherichia coli* [16]. Similar studies led to the identification of antimicrobial and antimycobacterial domains in bovine NK-lysin as well [11, 17]. In addition, the secretion of granzymes, granulysin, and perforin has been shown to be effective against intracellular parasites such as *Trypanosoma cruzi*, *Toxoplasma gondii*, and *Leishmania major* [18]. Although NK-lysins and granulysin can efficiently kill extracellular bacteria, both proteins need the help of perforin to enter cells to kill intracellular pathogens [12, 18]. Additionally, granulysin also shows immunomodulatory properties by activating monocytes to secrete chemokines to attract other cells to the site of inflammation, as well as proinflammatory cytokines [19]. Mechanisms of innate and adaptive immunity have been well defined for many mycobacterial pathogens, but there is a lack of information regarding the potential bactericidal activity of NK-lysins, particularly for cattle.

Unlike the human and pig genomes, the cattle genome contains four different bNK-lysin genes (*NK1*, *NK2A*, *NK2B*, and *NK2C*) [17]. All four bNK-lysin-derived peptides are functional and show strong antimicrobial activity against various bacterial pathogens involved in bovine respiratory diseases, such as *Mycobacterium bovis*, *Histophilus somni*, and *Mycoplasma bovis* [11, 17, 20]. Therefore, we hypothesize that bNK-lysin peptides are active against MAP as well. In the present study, we describe the antimicrobial activity of all four bNK-lysin peptides against MAP.

All four bNK-lysin peptides (bNK1, bNK2A, bNK2B, and bNK2C) were chemically synthesized as 30-mer peptides corresponding to the functional region helix2-loop-helix3 (Peptide 2.0 Inc, Chantilly, VA, USA) as trifluoroacetate salt (95% purity) [20, 21]. For cell culture experiments with a monocyte-derived macrophage infection model, the bNK2A peptide N-terminally fused to a cell-penetrating peptide derived from transactivator of transcription of human immunodeficiency virus type-1 (TAT_47-57_, TAT-bNK2A) was similarly synthesized, promoting entrance of bNK2A into the cell [22].

The direct antimicrobial killing activity of bNK-lysin peptides was assessed by incubating with MAP in culture medium in 3 replicate experiments. An isolate of MAP (strain 167) from a cow with clinical disease was grown to log phase (OD_540nm_ = 0.2–0.4) at 39 °C in Middlebrook 7H9 broth (M7H9; Becton Dickinson, Franklin Lakes, NJ) as previously described [23], and aliquots were stored at −80 °C. Briefly, 50 µL of diluted MAP (~1 × 10^5^ CFU) was incubated with 50 μL of bNK-lysin peptides in M7H9 to achieve final concentrations of 10, 50, or 100 µM in 96-well plates [11]. MAP incubated with M7H9 served as a negative control. The plate was covered, sealed in a Ziploc bag, and incubated at 39 °C for 72 h with gentle rocking. Viable MAP was identified using a novel propidium monoazide (PMAxx™, Biotium, Fremont, CA, USA) coupled with quantitative PCR (qPCR; PMA-qPCR) using IS900 gene-specific primers and probes with extracted genomic DNA [24–28]. Briefly, the bacteria were transferred to microfuge tubes, pelleted by centrifugation (10 000 *g* for 10 min), and then resuspended in 25 µM PMAxx solution. Samples were incubated at RT for 10 min in the dark, placed in a PMA-Lite LED Photolysis Device and exposed to LED light for 15 min, allowing for DNA-PMAxx crosslinking. Thereafter, genomic DNA (gDNA) was extracted from MAP pellets using the DNeasy^®^ Blood and Tissue Kit according to the manufacturer’s instructions (Qiagen Inc, Valencia, CA, USA). qPCR was performed on extracted gDNA using MAP-specific primers (forward: 5′-CCGCTAATTGAGAGATGCGATTGG-3′; reverse: 5′-AATCAACTCCAGCAGCGCGGCCTCG-3′) and a probe (5′-FAM-TCCACGCCCGCCCAGACAGG-TAMRA-3′) to the IS900 gene target as described previously [24]. PMA-qPCR was conducted in an Applied Biosystems 7500 fast real-time PCR system (Life Technologies) by using TaqMan^®^ Fast Advanced Master Mix (Life Technologies). The PMA-qPCR assay was performed under the following conditions: 1 cycle at 95 °C for 10 min followed by 40 cycles of denaturation at 94 °C for 25 s and annealing-extension at 60 °C for 1 min. The total qPCR reaction volume was 25 µL, with 5 µL of sample, 12.5 µL of Master Mix (Life Technologies), 6.88 µL of ultrapure distilled DNase- and RNase-free water, 200 nM of each primer, and 100 nM of probe. Each qPCR plate also contained a standard curve ranging from 1 ng/µL to 1 fg/µL that was generated from MAP strain 167 genomic DNA, no-template negative controls, and a positive control of MAP strain 19,698 genomic DNA. The mean cycle threshold (Ct) values of triplicate wells were used to calculate the frequency of viable MAP present in each sample. The percentage of remaining viable MAP in bNK-lysin peptide-treated samples was calculated using the following equation ([average number of live MAP in peptide-treated sample/average number of live MAP in the negative control] × 100). Ct values are inversely proportionate to the viable MAP in the tested samples. The final percentages of live or dead MAP in each peptide treatment are based on three independent experiments.

The physical impacts of bNK-lysin on MAP were visualized by transmission electron microscopy (TEM). Fifty microlitres of MAP (~5 × 10^6^ CFU) was transferred into a 96-well plate and incubated with either 50 μL of bNK2A (100 μM concentration) or M7H9 at 39 °C for 72 h. Samples were fixed with 3% glutaraldehyde and processed for TEM as described previously [29]. Bacterial pellets were then rinsed in cacodylate buffer, postfixed in 1% osmium tetroxide, dehydrated in a graded series of ethanol (30–100%, ~6 h), washed with 100% propylene oxide (~1.5 h), and embedded in EMbed-812 resin (Electron Microscopy Sciences, Hatfield, PA) for ~36 h. Sections of the bacterial pellets were cut and stained with uranyl acetate and lead citrate. Sections were examined with an FEI Tecnai G2 Biotwin (ThermoFisher Scientific, Carlsbad, CA, USA) TEM, and images were captured with an Advanced Microscopy Technologies (AMT Inc., Danvers, MN, USA) imaging camera. TEM was repeated twice with three replicates for each sample.

The bactericidal activity of bNK2A lysin was further assessed in a cell culture system. Assay conditions were initially optimized using monocyte-derived macrophages (MDMs) prepared from two cows. For the final experiments, blood was collected from four healthy cows, and PBMCs were isolated as described previously [30]. Briefly, MDMs were prepared by plating PBMCs onto ibiTreat μ-slides (Ibidi USA, Inc., Fitchburg, WI, USA). On day 6, MAP was diluted in RPMI-1640 medium with 1% foetal bovine serum (FBS) and added to MDMs at an MOI of 10:1. After 2 h of incubation, bNK2A or TAT-bNK2A peptides (10 μM) were added into wells with MAP and further incubated for 24 h. Assay controls were MDMs alone and MDMs infected with only MAP. Live and dead intracellular MAP were identified by SYTO 9 and propidium iodide (PI) using a *Bac*Light bacterial viability kit (Cat# L7012, ThermoFisher Scientific, Carlsbad, CA, USA) and 0.1% saponin as previously described [31]. MDMs were incubated with SYTO 9 (5 μM) and PI (30 μM) at room temperature in the dark for 15 min. Media containing the diluted dyes were then removed from the wells and replaced with MOPS buffer. Macrophages were stained using anti-bovine CD68 (Clone EBM11, Agilent-DAKO, Santa Clara, CA, USA) followed by Alexa Fluor 647 (AF647)-conjugated secondary antibody (ThermoFisher Scientific) by incubating in the dark at room temperature for 1 h each. Imaging was performed with a Nikon A1R + Confocal Laser Scanning Microscope System (Nikon Instrument, Melville, NY, USA). Calibration was performed for the dyes using the software, and sequentially collected frames of individual channels were merged and then saved as ND2 files. Images were obtained with a plan Fluor 40 × objective lens (oiled) at numerical aperture 1.3. At least 10 images per treatment group were collected for analyses. Binary layers for each channel (SYTO 9, 488 nm; PI, 561 nm; AF647 640 nm) facilitated automated detection of live and dead MAP within CD68^+^ regions of interest (ROIs) representing independent MDMs within the images.

Two statistical analyses were performed using the MIXED procedure from SAS (SAS Inc., Cary, North Carolina, USA). The first analysis included the fixed effects of concentration (10, 50, and 100 µM), experiment (experiments 1 and 2), and bNK-lysin peptides (bNK1, bNK2A, bNK2B, and bNK2C). The second analysis included the fixed effects of MAP (dead or alive), the bNK-lysin peptides (control, bNK2A, and TAT-bNK2A), and the interaction between MAP and bNK-lysin peptides. Mean comparisons were performed using the predicted differences (PDIFF) option from the MIXED procedure. The term “significant” indicates a value of *P* < 0.05.

**Figure 1:**
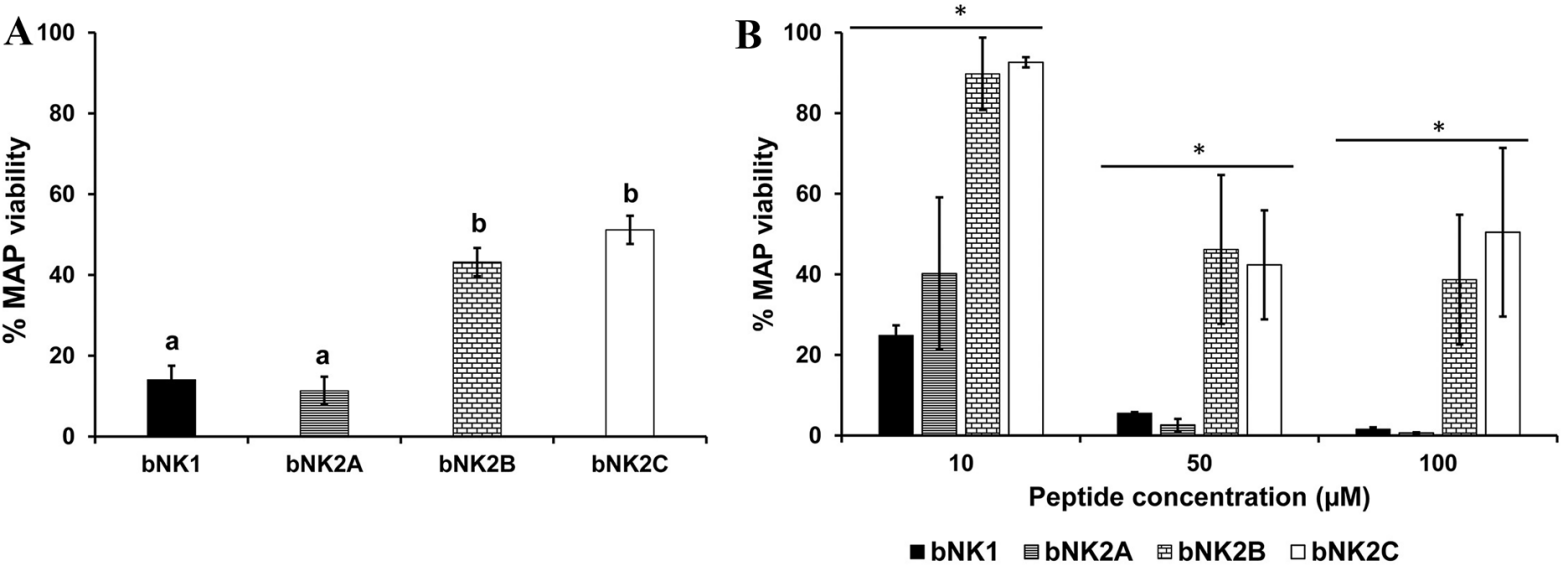
**Antimicrobial activity of bNK-lysin peptides against *M. paratuberculosis.*** The remaining viable MAP in bNK-lysin peptide-treated samples was assessed by PMA-qPCR assay. Mean percentages of viable MAP with standard error of means in the overall (**A**) and concentration-dependent (**B**) MAP killing assay with bNK-lysin peptides are shown in the graphs. ^a^Statistically different (*P* ≤ 0.002; ^b^Statistically different (*P* < 0.0001); *P < 0.0001.

MAP is a very slow-growing bacterium that takes up to 12 weeks to form visible colonies on agar growth medium but also clumps readily, making quantification difficult. Therefore, a newer methodology using a propidium monoazide-based qPCR viability assay that has previously been successfully used to determine viable MAP [25–29] was used to determine the antimicrobial activity of bNK-lysin peptides against MAP. The anti-MAP activity of all four bNK-lysin peptides was significantly different from that of the control (Figure 1A; *P* < 0.0001). Concentration-dependent MAP killing activity was observed for all four bNK-lysin peptides during a 72 h incubation period (Figure 1B; *P* < 0.0001). Although bNK1 and bNK2A were highly active against MAP at the concentrations of 50 and 100 µM, with less than 6% viable MAP remaining in the samples, approximately 30% and 35% viable MAP could still be detected in bNK2B- and bNK2C-treated samples (Figure 1B). Additionally, reduced antimicrobial activity, as indicated by higher percentages of remaining viable MAP, was detected with bNK2B- and bNK2C-treated samples (~85% and 93% viability) compared to bNK1- and bNK2A-treated samples (~25% and 34% viability) at the final peptide concentration of 10 µM (Figure 1B).

**Figure 2:**
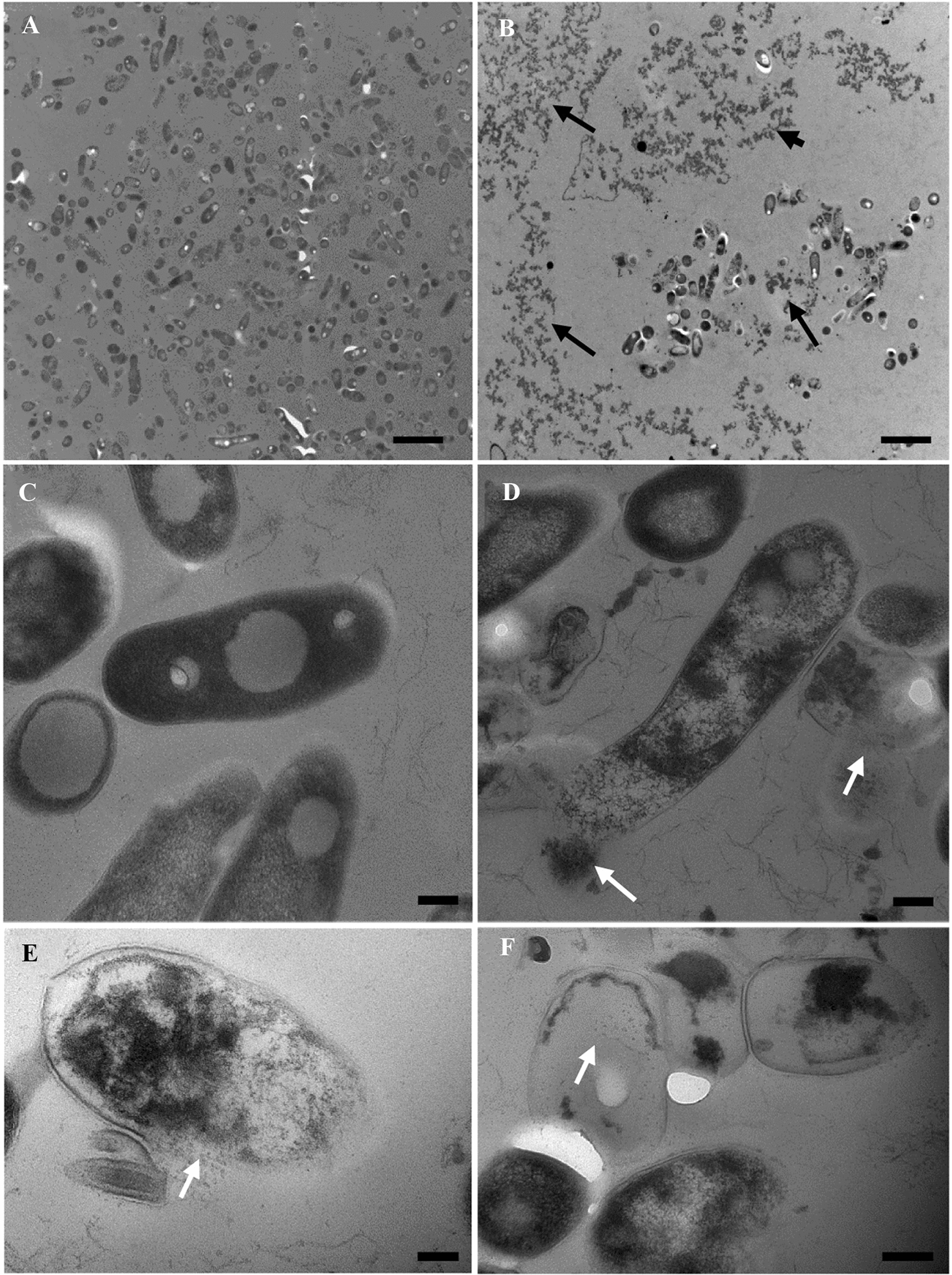
**Membranolytic activity of the bNK2A peptide against *M. paratuberculosis.*** (**A**) MAP control (× 4800, scale bar = 2 µm); (**B**) MAP with 100 µM bNK2A (cell debris is indicated by black arrows; × 4800, scale bar = 2 µm); (**C**) MAP control (× 68 000, scale bar = 200 nm); (**D**) MAP with 100 µM bNK2A (intracellular content leakage is indicated by white arrows; × 68 000, scale bar = 200 nm); (**E**) MAP with 100 µM bNK2A (intracellular content leakage is indicated by a white arrow; × 98 000, scale bar = 100 nm); (**F**) MAP with 100 µM bNK2A (ghost cell is indicated by a white arrow; × 68 000, scale bar = 200 nm).

Since the bNK2A peptide showed the highest in vitro MAP killing activity, this peptide was selected as a model bNK-lysin peptide to visualize ultrastructural changes in MAP. After 72 h of incubation, both control and bNK2A-treated (100 µM) samples were processed for TEM. Compared to the control samples (Figure 2A), bNK2A-treated samples showed fewer MAP and abundant cell debris (black arrows) (Figure 2B). The presence of bacteria with intact membranes along with electron-dense cytoplasm suggested that most of the MAP in the control samples was viable (Figure 2C). Conversely, bacteria with damaged inner and outer membranes (Figure 2D, E) with reduced cytoplasmic density due to the leakage of intracellular contents (white arrows, Figure 2D, E) and cell debris were visible in bNK2A-treated samples, indicating that the majority of MAP were dead. Several MAP devoid of cytoplasmic contents (ghost cells) were also found in bNK2A-treated samples (Figure 2F, white arrow).

**Figure 3:**
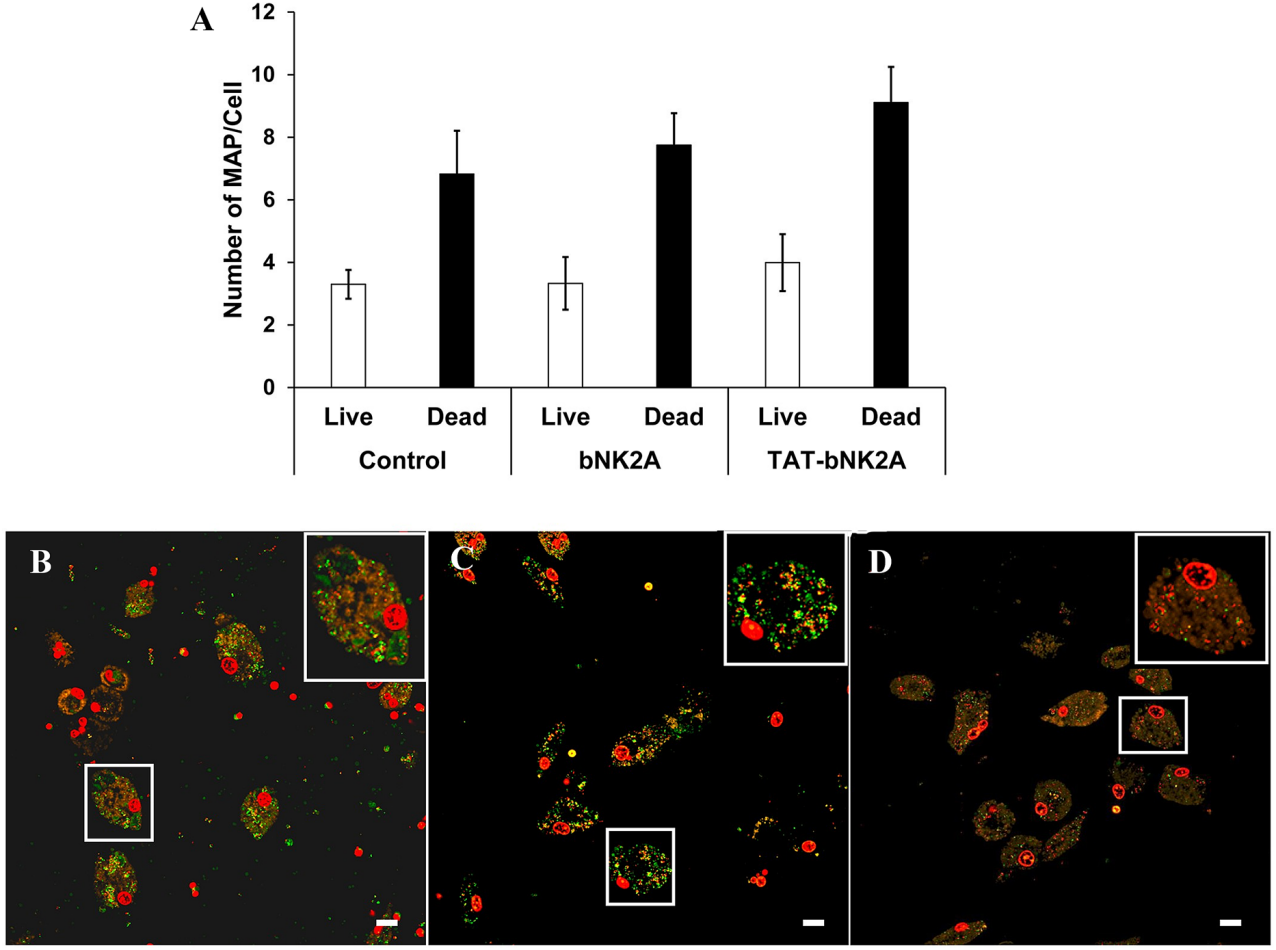
**Antimicrobial activity of TAT-bNK2A and bNK2A against intracellular *M. paratuberculosis.*** (**A**) Mean percentages of live and dead intracellular MAP in MDMs with the corresponding standard error of the mean. Representative confocal laser scanning microscopy images of live (green) and dead (red) intracellular MAP in (**B**) control (**C**) 10 µM bNK2A and (**D**) 10 µM TAT-bNK2A peptide-treated MDMs. Large red circles within MDMs are PI-stained nuclei. The identification of MDMs in each well was confirmed by positive labelling with anti-CD68 antibody (orange colour cells, B, C and D). Selected MDMs (small white boxes) in each panel were digitally enlarged and are shown in the upper right corner (large white boxes). Means and SEMs of live and dead MAP in infected MDMs were calculated from four cows. (Scale bar = 2 µm). *P* = 0.6923.

An MDM infection model was utilized to assess the effectiveness of bNK2A in penetrating macrophages and attaining access to MAP. Since cell-penetrating peptides (CPPs) are known to translocate through the plasma membrane without causing significant damage to the membranes [22], a chimaeric bNK2A peptide N-terminally fused to a well-characterized CPP transactivator of transcription of HIV was also synthesized (TAT-bNK2A). Since both peptides at the concentrations of 50 and 100 µM were cytotoxic to MDMs (Additional file 1), we only tested the 10 µM concentration of both peptides in the intracellular MAP killing assay. MDMs infected with MAP were treated with bNK2A or TAT-bNK2A peptides for 24 h. Confirmation of MDMs within the slides was performed by CD68 antibody labelling (orange colour cells in Figure 3B–D). Approximately two-fold more dead MAP (per cell) than live MAP (per cell) was observed for all three treatment groups, indicating the ability of MDMs to kill intracellular MAP (Figure 3A). Although the number of dead MAP was not significantly different among the three samples (*P* = 0.6923), slightly improved MAP killing activity was observed upon treatment of MDMs with TAT-bNK2A (Figure 3A). Compared to controls, TAT-bNK2A showed ~25% improvement in intracellular MAP killing.

## Discussion

The sensitivity of *Mycobacterium bovis* to bNK2B and bNK2C has been reported previously [11]. However, studies with any of the four bNK-lysin peptides have not been reported for other mycobacterial pathogens, including MAP. Therefore, the goal of the present study was to assess the antimicrobial activity of all four bNK-lysin peptides against MAP. A novel PMA-qPCR technique has been successfully used to determine the viability of various bacterial pathogens, including MAP [25–28]. This assay is very helpful to determine the viability of the remaining MAP following incubation with peptides since MAP can take up to 12 weeks to form visible colonies in solid medium. PMA, a fluorescent dye that indicates cell membrane impairment, can only enter bacteria with damaged or compromised membranes. Upon photolysis with LED light, PMA binds covalently to DNA and prevents DNA amplification by PCR. Thus, only live bacterial DNA can be amplified by PMA-qPCR. The results of the PMA-qPCR viability assay suggested that although MAP was highly sensitive to all four bNK-lysin-mediated killing at higher peptide concentrations, bNK1 and bNK2A were most effective, with up to 94% MAP killing. The strong bactericidal activity of bNK-lysin peptides has previously been described for multiple extracellular gram-positive and gram-negative bacterial species [17, 20, 21, 32]. Importantly, most of the bacteria tested were more sensitive to bNK-lysin peptides at lower micromolar concentrations (< 5 µM) than what was shown to be effective for mycobacteria, suggesting that bNK-lysin peptides are extremely effective under in vitro assay conditions. In contrast, a higher concentration (100 µM) of two tested bNK-lysin peptides (bNK2B and bNK2C) was required to show anti-*M. bovis* activity, with ~70% killing observed at that concentration [11]. The TEM results with the bNK2A peptide showed dead MAP with damaged membranes, intracellular content leakage and ghost cells (completely devoid of intracellular contents). Similar to other AMPs, our observations clearly suggest that MAP killing is due to the membranolytic activity of bNK-lysin peptides. Unlike many other bacterial species, the thick cell wall of MAP is composed of more than 60% lipids, demonstrating a high level of resistance to heat and chemical treatment, and can contribute to the survival of MAP within host macrophages [2–5]. Therefore, the concentration of bNK-lysin peptide required to kill MAP may be attributed to the difficulty of producing membrane pores due to the higher lipid content of the MAP cell wall.

Bacterial cell membranes are negatively charged due to the presence of lipopolysaccharides in gram-negative bacteria or teichoic/lipoteichoic acids in gram-positive bacteria. Although the mycobacterial membrane is different from other bacteria due to the presence of an unusual amount of lipids such as mycolic acids, mycobacterial membranes are also negatively charged [33]. Cationic AMPs electrostatically interact with negatively charged bacterial surfaces, resulting in the insertion of AMPs into the membrane and the production of pores by barrel-starve, carpet or toroidal-pore mechanisms leading to bacterial death [8]. The bNK-lysin peptides synthesized for the present study are cationic peptides due to the presence of several arginine and lysine residues. bNK1 has the lowest net charge of + 5.0, and bNK2A has the highest net charge of + 7.9, while bNK2B and bNK2C have net charges of + 6.9 and + 5.9, respectively. Therefore, we expected all four bNK-lysin peptides to show strong electrostatic interactions with MAP membranes followed by pore formation. It is not clear why we observed lower anti-MAP activity of bNK2B and bNK2C herein, despite having higher net positive charges compared to bNK1.

In general, mycobacteria are facultative intracellular pathogens that reside within macrophages in the host. *M. tuberculosis* is known to avoid phagosome-lysosome fusion pathways and resides inside unique phagosomes [34]. MAP is also known to survive inside macrophages of the host. Studies with the murine macrophage J774 cell line suggested that similar to *M. tuberculosis*, MAP can also prevent phagosome-lysosome maturation and/or phagosome acidification [35, 36]. Previously, we demonstrated biphasic effects of MAP killing in the J774 macrophage cell line, with MAP numbers increasing during the first 24 h followed by rapid decreases in MAP in the subsequent 24–72 h period [29]. Since macrophages seemed less able to control MAP replication in the first 24 h of infection, our efforts focused on the evaluation of bNK2A peptides in that critical time period. Since MDMs were sensitive to bNK-lysins at 50–100 µM concentrations, resulting in cell death, only the final peptide concentration of 10 µM was tested with MAP-infected MDMs. Although antimicrobial proteins such as human granulysin can efficiently kill various extracellular bacteria [16], a previous study reported that granulysin was unable to kill intracellular *M. tuberculosis* without coincubation with perforin [12]. In this study, we selected a well-characterized cell-penetrating TAT peptide fused with bNK2A (TAT-bNK2A) to improve the delivery of bNK2A into MDMs [22]. Although greater anti-MAP activity was expected after treatment of MDMs with the TAT-bNK2A peptide, it showed only a 25% improvement in intracellular MAP killing compared to controls during 24 h of incubation. In experiments with direct application of bNK-lysins to MAP in culture, the lowest concentration (10 µM) of bNK2A was bactericidal, demonstrating ~66% MAP killing during 72 h of incubation, but the killing was attenuated compared to higher concentrations. It is clear that a higher concentration of peptide is probably needed to effectively kill MAP in the macrophage model, but this is hindered by detrimental effects on the macrophage itself. Fusion of bNK2A with the TAT peptide improved intracellular MAP killing by allowing lysin to gain entrance into the cell more easily; however, further optimization is needed to improve MAP killing by macrophages without adverse effects.

In conclusion, bNK-lysin peptides (bNK2A) enhanced the killing of MAP, both intracellularly and extracellularly. These results suggest that bNK-lysins may play an important role in the initial defence against MAP before and after it is taken up by resident phagocytes in the intestine of affected animals.

## Supplementary Information

**Additional file 1. Cytotoxic effect of the bNK2A peptide on monocyte-derived macrophages (MDMs).** (A) Control, (B) 10 µM and (C) 50 µM bNK2A-treated MDMs were incubated at 39 °C for 24 h. Dead cells were identified by staining with PI (scale bar = 20 µm).

## Abbreviations

AMPs: Antimicrobial peptides
bNK-lysins: Bovine NK-lysins
CPP: Cell-penetrating peptides;
FBS: Foetal bovine serum
MAP: *Mycobacterium avium* Subspecies *paratuberculosis*
M7H9: Middlebrooks 7H9 medium
MDMs: Monocyte-derived macrophages
PMA-qPCR: Propidium monoazide-coupled viability quantitative polymerase chain reaction
TAT: Transactivator of transcription of human immunodeficiency virus type 1
TEM: Transmission electron microscopy

## References

[1] Sweeney RW (1996). Transmission of paratuberculosis. Vet Clin North Am Food Anim Pract.

[2] Rowe MT, Grant IR (2006). *Mycobacterium avium* ssp. paratuberculosis and its potential survival tactics. Lett Appl Microbiol.

[3] Bansal-Mutalik R, Nikaido H (2014). Mycobacterial outer membrane is a lipid bilayer and the inner membrane is unusually rich in diacyl phosphatidylinositol dimannosides. Proc Natl Acad Sci USA.

[4] Gao A, Mutharia L, Chen S, Rahn K, Odumeru J (2002). Effect of pasteurization on survival of Mycobacterium paratuberculosis in milk. J Dairy Sci.

[5] Sung N, Collins MT (1998). Thermal tolerance of Mycobacterium paratuberculosis. Appl Environ Microbiol.

[6] Zhai W, Wu F, Zhang Y, Fu Y, Liu Z (2019). The immune escape mechanisms of Mycobacterium Tuberculosis. Int J Mol Sci.

[7] Tessema MZ, Koets AP, Rutten VP, Gruys E (2001). How does *Mycobacterium avium* subsp. paratuberculosis resist intracellular degradation?. Vet Q.

[8] Brogden KA (2005). Antimicrobial peptides: pore formers or metabolic inhibitors in bacteria?. Nat Rev Microbiol.

[9] Sierra JM, Fuste E, Rabanal F, Vinuesa T, Vinas M (2017). An overview of antimicrobial peptides and the latest advances in their development. Expert Opin Biol Ther.

[10] Bahar AA, Ren D (2013). Antimicrobial peptides. Pharmaceuticals.

[11] Endsley JJ, Furrer JL, Endsley MA, McIntosh MA, Maue AC, Waters WR, Lee DR, Estes DM (2004). Characterization of bovine homologues of granulysin and NK-lysin. J Immunol.

[12] Stenger S, Hanson DA, Teitelbaum R, Dewan P, Niazi KR, Froelich CJ, Ganz T, Thoma-Uszynski S, Melian A, Bogdan C, Porcelli SA, Bloom BR, Krensky AM, Modlin RL (1998). An antimicrobial activity of cytolytic T cells mediated by granulysin. Science.

[13] Andersson M, Gunne H, Agerberth B, Boman A, Bergman T, Sillard R, Jornvall H, Mutt V, Olsson B, Wigzell H (1995). NK-lysin, a novel effector peptide of cytotoxic T and NK cells. Structure and cDNA cloning of the porcine form, induction by interleukin 2, antibacterial and antitumour activity. EMBO J.

[14] Pena SV, Hanson DA, Carr BA, Goralski TJ, Krensky AM (1997). Processing, subcellular localization, and function of 519 (granulysin), a human late T cell activation molecule with homology to small, lytic, granule proteins. J Immunol.

[15] Munford RS, Sheppard PO, O'Hara PJ (1995). Saposin-like proteins (SAPLIP) carry out diverse functions on a common backbone structure. J Lipid Res.

[16] Andreu D, Carreno C, Linde C, Boman HG, Andersson M (1999). Identification of an anti-mycobacterial domain in NK-lysin and granulysin. Biochem J.

[17] Chen J, Huddleston J, Buckley RM, Malig M, Lawhon SD, Skow LC, Lee MO, Eichler EE, Andersson L, Womack JE (2015). Bovine NK-lysin: Copy number variation and functional diversification. Proc Natl Acad Sci USA.

[18] Dotiwala F, Mulik S, Polidoro RB, Ansara JA, Burleigh BA, Walch M, Gazzinelli RT, Lieberman J (2016). Killer lymphocytes use granulysin, perforin and granzymes to kill intracellular parasites. Nat Med.

[19] Deng A, Chen S, Li Q, Lyu SC, Clayberger C, Krensky AM (2005). Granulysin, a cytolytic molecule, is also a chemoattractant and proinflammatory activator. J Immunol.

[20] Dassanayake RP, Falkenberg SM, Briggs RE, Tatum FM, Sacco RE (2017). Antimicrobial activity of bovine NK-lysin-derived peptides on bovine respiratory pathogen Histophilus somni. PLoS ONE.

[21] Dassanayake RP, Falkenberg SM, Register KB, Samorodnitsky D, Nicholson EM, Reinhardt TA (2018). Antimicrobial activity of bovine NK-lysin-derived peptides on *Mycoplasma bovis*. PLoS ONE.

[22] El-Andaloussi S, Jarver P, Johansson HJ, Langel U (2007). Cargo-dependent cytotoxicity and delivery efficacy of cell-penetrating peptides: a comparative study. Biochem J.

[23] Stabel JR, Bradner L, Robbe-Austerman S, Beitz DC (2014). Clinical disease and stage of lactation influence shedding of *Mycobacterium avium* subspecies paratuberculosis into milk and colostrum of naturally infected dairy cows. J Dairy Sci.

[24] Leite FL, Stokes KD, Robbe-Austerman S, Stabel JR (2013). Comparison of fecal DNA extraction kits for the detection of *Mycobacterium avium* subsp. paratuberculosis by polymerase chain reaction. J Vet Diagn Invest.

[25] Kralik P, Nocker A, Pavlik I (2010). *Mycobacterium avium* subsp. paratuberculosis viability determination using F57 quantitative PCR in combination with propidium monoazide treatment. Int J Food Microbiol.

[26] Kralik P, Babak V, Dziedzinska R (2018). The impact of the antimicrobial compounds produced by lactic acid bacteria on the growth performance of *Mycobacterium avium* subsp. paratuberculosis. Front Microbiol.

[27] Ricchi M, De Cicco C, Kralik P, Babak V, Boniotti MB, Savi R, Cerutti G, Cammi G, Garbarino C, Arrigoni N (2014). Evaluation of viable *Mycobacterium avium* subsp. paratuberculosis in milk using peptide-mediated separation and propidium monoazide qPCR. FEMS Microbiol Lett.

[28] Pribylova R, Kubickova L, Babak V, Pavlik I, Kralik P (2012). Effect of short- and long-term antibiotic exposure on the viability of *Mycobacterium avium* subsp. paratuberculosis as measured by propidium monoazide F57 real time quantitative PCR and culture. Vet J.

[29] Bannantine JP, Stabel JR (2002). Killing of *Mycobacterium avium* subspecies paratuberculosis within macrophages. BMC Microbiol.

[30] Khalifeh MS, Stabel JR (2004). Effects of gamma interferon, interleukin-10, and transforming growth factor beta on the survival of *Mycobacterium avium* subsp. paratuberculosis in monocyte-derived macrophages from naturally infected cattle. Infect Immun.

[31] Lazaro-Diez M, Chapartegui-Gonzalez I, Redondo-Salvo S, Leigh C, Merino D, Segundo DS, Fernández A, Navas J, Icardo JM, Acosta F, Ocampo-Sosa A, Martínez-Martínez L, Ramos-Vivas J (2017). Human neutrophils phagocytose and kill *Acinetobacter baumannii* and *A. pittii*. Sci Rep.

[32] Chen J, Yang C, Tizioto PC, Huang H, Lee MO, Payne HR, Lawhon SD, Schroeder F, Taylor JF, Womack JE (2016). Expression of the Bovine NK-lysin gene family and activity against respiratory pathogens. PLoS ONE.

[33] Bendinger B, Rijnaarts HH, Altendorf K, Zehnder AJ (1993). Physicochemical cell surface and adhesive properties of coryneform bacteria related to the presence and chain length of mycolic acids. Appl Environ Microbiol.

[34] Vergne I, Fratti RA, Hill PJ, Chua J, Belisle J, Deretic V (2004). Mycobacterium tuberculosis phagosome maturation arrest: mycobacterial phosphatidylinositol analog phosphatidylinositol mannoside stimulates early endosomal fusion. Mol Biol Cell.

[35] Kuehnel MP, Goethe R, Habermann A, Mueller E, Rohde M, Griffiths G, Valentin-Weigand P (2001). Characterization of the intracellular survival of *Mycobacterium avium* ssp. paratuberculosis: phagosomal pH and fusogenicity in J774 macrophages compared with other mycobacteria. Cell Microbiol.

[36] Hostetter J, Steadham E, Haynes J, Bailey T, Cheville N (2003). Phagosomal maturation and intracellular survival of *Mycobacterium avium* subspecies paratuberculosis in J774 cells. Comp Immunol Microbiol Infect Dis.

