# Supplementary figures and images for: Bovine NK-lysin-derived peptides have bactericidal effects against Mycobacterium avium subspecies paratuberculosis
Source: Vet Res. 2021 Jan 21;52:11. doi: 10.1186/s13567-021-00893-2 (PMC7818946; doi:10.1186/s13567-021-00893-2)

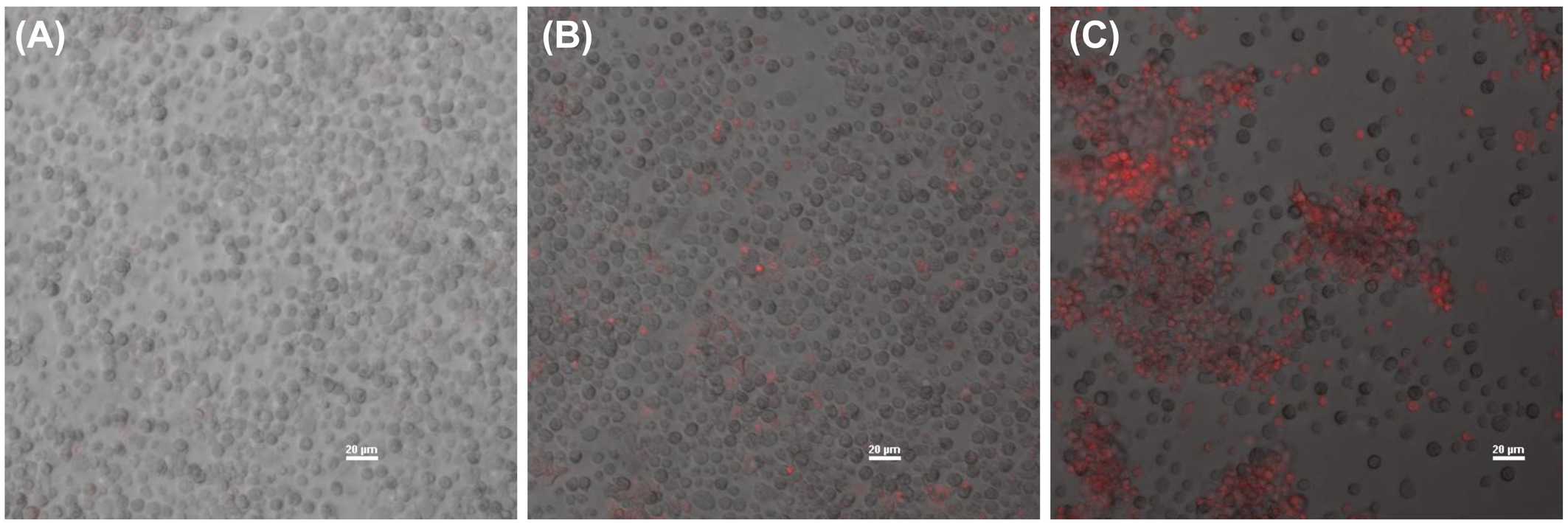

Supplement: Supplementary file 1 — Additional file 1. Cytotoxic effect of the bNK2A peptide on monocyte-derived macrophages (MDMs). (A) Control, (B) 10 µM and (C) 50 µM bNK2A-treated MDMs were incubated at 39 °C for 24 h. Dead cells were identified by staining with PI (scale bar = 20 µm). [file 13567_2021_893_MOESM1_ESM.tiff]
